# Innovative Cell-Based Therapies and Conditioning to Cure RAG Deficiency

**DOI:** 10.3389/fimmu.2020.607926

**Published:** 2020-11-19

**Authors:** Anna Villa, Valentina Capo, Maria Carmina Castiello

**Affiliations:** ^1^ San Raffaele Telethon Institute for Gene Therapy (SR-Tiget), IRCCS San Raffaele Scientific Institute, Milan, Italy; ^2^ Istituto di Ricerca Genetica e Biomedica, Consiglio Nazionale delle Ricerche (IRGB-CNR), Milan, Italy

**Keywords:** ****RAG genes, gene therapy, non-genotoxic conditioning, severe combined immunodeficiency, hematopoietic stem cell transplantation, Omenn syndrome, leaky SCID

## Abstract

Genetic defects in recombination activating genes (RAG) 1 and 2 cause a broad spectrum of severe immune defects ranging from early severe and repeated infections to inflammation and autoimmune manifestations. A correlation between *in vitro* recombination activity and immune phenotype has been described. Hematopoietic cell transplantation is the treatment of care; however, the availability of next generation sequencing and whole genome sequencing has allowed the identification of novel genetic RAG variants in immunodeficient patients at various ages, raising therapeutic questions. This review addresses the recent advances of novel therapeutic approaches for RAG deficiency. As conventional myeloablative conditioning regimens are associated with acute toxicities and transplanted-related mortality, innovative minimal conditioning regimens based on the use of monoclonal antibodies are now emerging and show promising results. To overcome shortage of compatible donors, gene therapy has been developed in various RAG preclinical models. Overall, the transplantation of autologous gene corrected hematopoietic precursors and the use of non-genotoxic conditioning will open a new era, offering a cure to an increasing number of RAG patients regardless of donor availability and severity of clinical conditions.

## Introduction

Effective adaptive immunity relies on the ability of T and B lymphocytes to express the vast majority of antigen receptors. During differentiation, T and B cells assemble T cell antigen receptor (TCR) and B cell receptor (BCR) respectively, by a complex process named V(D)J recombination that recognizes each segment of V, D and J flanked by recombination signal sequences (RSSs) (1). The recombination activating genes (*RAG*) 1 and 2, the first players of this molecular process, form a complex and introduce a DNA double strand break (DSB) in the RSSs giving rise to a diverse repertoire of antigen specific receptors. Null mutations in *RAG* genes cause an arrest at DN3 and pre-B1 stage of T and B cell development respectively (2). The T^-^ B^-^ natural killer (NK)^+^ severe combined immunodeficiency (SCID) phenotype is characterized by repeated severe infections caused by common viral pathogens and opportunistic pathogens that lead to death in the absence of hematopoietic stem cell transplantation (HSCT), which represents the treatment of choice (3).

Missense mutations impairing RAG functions while permitting occasional recombination activity lead to the generation and expansion of oligoclonal T cell population. Extensive molecular studies have defined the biochemical effect of amino acid changes on the recombination activity, providing evidence that residual activity sustained at least by one allele can lead to a peculiar immune phenotype named Omenn syndrome (OS) (4, 5). These patients present severe erythroderma, lymphadenopathy with hepatosplenomegaly, colitis, repeated infections and inflammatory pneumonitis. Activated oligoclonal and autoreactive T cells circulate in the peripheral blood and tend to migrate to the gut and skin mainly contributing to tissue damage that correlates with the severity of the disease (6, 7). IgM, IgA and IgG are usually absent or barely detectable in the serum but elevated IgE levels are found despite lack of circulating B cells (8, 9). Recently, next generation sequence identified new forms of RAG-SCID presenting with a milder phenotype than the classical signs of Omenn syndrome (10, 11). The description of these cases has further broadened the spectrum of clinical manifestations caused by RAG mutations, posing diagnostic and therapeutic questions (12, 13).

Leaky or atypical SCID (AS) patients harbor missense mutations and in the majority of these patients the diagnosis is delayed, with RAG mutations identified in childhood (median 5 years) or even in adolescent or adult individuals (10, 13, 14). Of note, while autoimmune manifestations are rare in null SCID, cytopenia and autoimmune hemolytic anemia have been frequently reported in AS patients (15) and in some cases vasculitis resulting in digital necrosis have been reported (16). Other RAG-associated phenotypes include autoimmune cytopenia and oligoclonal expansion of TCR expressing γδ T cells in disseminated CMV infection (17, 18) or specific antibody and autoantibody production (19). Furthermore, Schuetz et al. reported granuloma formation in internal organs, skin, and mucous membranes in three unrelated females with severe viral infections and B cells lymphoma (20). Presence of the rubella virus vaccine strain has been demonstrated in the granulomas of some of these patients (21). This condition referred as “combined immunodeficiency with granuloma and/or autoimmunity” (CID-G/AI) may associate with autoimmune cytopenia and other autoimmune manifestations including myopathy and nephrotic syndrome (15). Finally, biallelic RAG mutations have been found in patients with idiopathic CD4 T cell lymphopenia (22), IgA deficiency (23, 24), hyper IgM syndrome (25) and impaired antibody production against polysaccharide antigens (24).

Extensive studies in mouse models have contributed to understand the *in vivo* effect of amino acid changes and the impact of RAG defects on the immune dysregulation (5, 11). Based on the evolutionary conservation of amino acid change identified in various clinical conditions, several groups have reproduced hypomorphic mutations in mouse models [reviewed in (11)]: the *Rag2^R229Q/R229Q^* mouse that fully recapitulates the clinical manifestations of OS (26); the spontaneous mouse mutant carrying a homozygous point mutation (R972Q) in the *Rag1* gene (27); the Rag1*^S723C/S723C^* showing profound B cell lymphopenia in the presence of significant serum levels of immunoglobulins and activated oligoclonal T cells (28). Novel mutants carrying mutations at C-terminal domain of *RAG1* and reproducing amino acid changes found in patients with CID-G/AI have been generated by gene editing (F971L, R972Q and R972W) corresponding to the human mutations (F974L, R975Q and R975W) (29). These models, associated with analysis of RAG patients, have allowed to dissect the defective mechanisms of central and peripheral tolerance and the contributive role of environment to the disease severity (7, 30). Parallel studies have identified the broad Th1/Th2/Th17 inflammatory signature, highlighting the complex scenario and its clinical implication (7). Therefore, these models have been exploited to assess the efficacy and safety of novel therapeutic strategies. Recent advances in conditioning regimens and availability of donor cells source have dramatically improved the outcome of HSCT that in case of null RAG forms was significantly worse than other forms of SCID (31).

**Figure 1:**
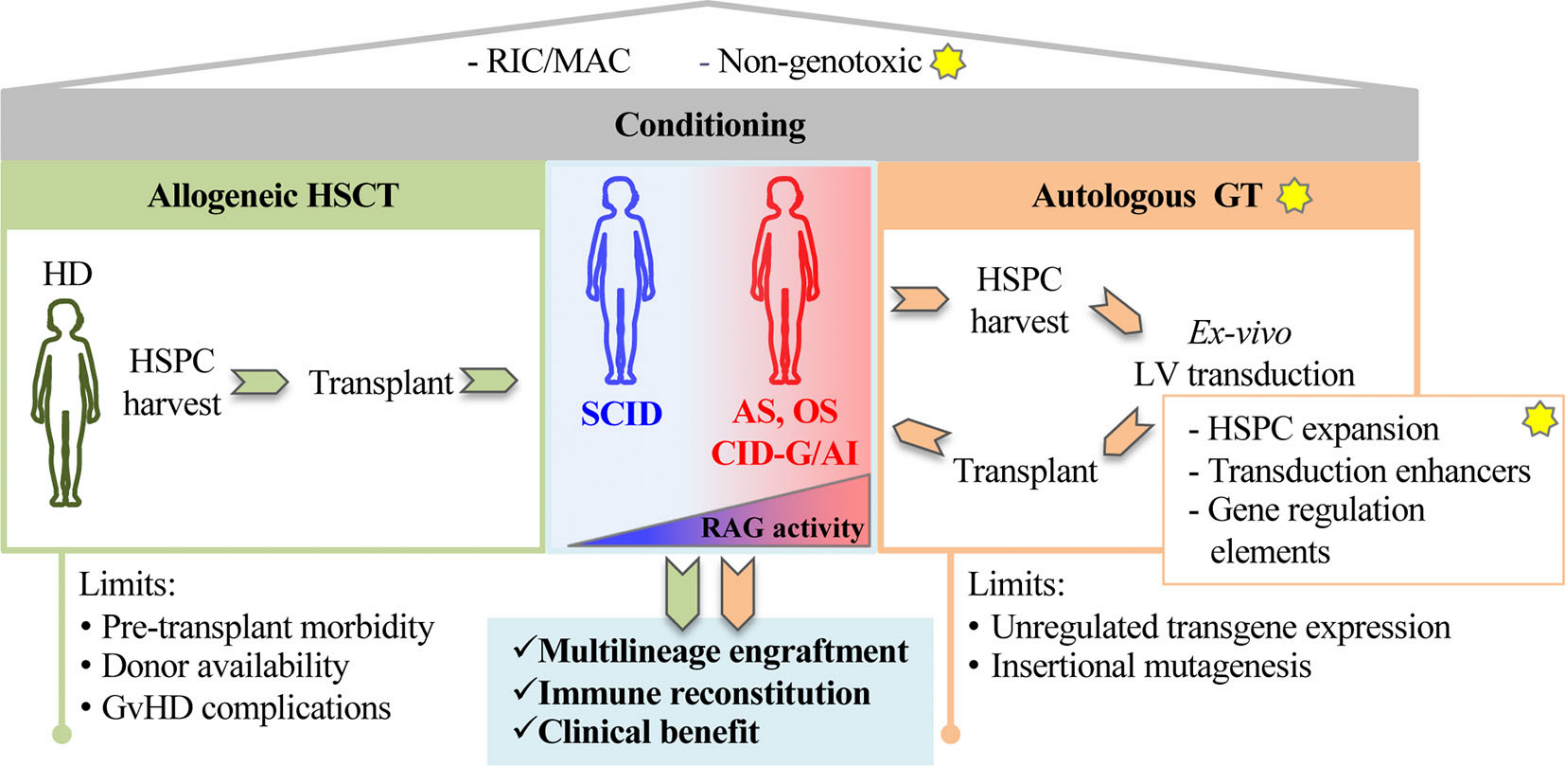
The figure shows principles of stem cell based gene therapy for recombination activating genes (RAG) disorders. The yellow stars indicate proposed innovative steps for the treatment of RAG-severe combined immunodeficiency (SCID). A preparative regimen as Reduced Intensity Conditioning (RIC) or myeloablative conditioning (MAC) is required to deplete endogenous hematopoietic precursors in RAG-deficient niches. Non-genotoxic compounds (anti-cKit mAb, anti-CD45 SAP) recently tested in preclinical models of RAG1 deficiencies represent a step forward toward new and safer form of conditioning. Patients carrying null RAG defect (SCID) or hypomorphic RAG defects (AS, OS, CID-G/AI) undergo conventional allogeneic hematopoietic stem cell transplantation (HSCT) (left green panel). Alternatively, autologous gene therapy (GT, right orange panel) CD34^+^ cells, isolated from bone marrow or mobilized peripheral blood, cultured and transduced in GMP conditions, are reinfused in the patient. New methods and reagents aimed at expanding HSPCs, enhancing transduction levels and regulating gene expression are being developed to boost GT efficiency. Limits of allogeneic HSCT and autologous GT are indicated at the bottom of the figure.

In RAG SCID, engraftment of donor cells requires myeloablative regimen, that eliminates arrested precursors fully occupying bone marrow and thymic niches. However, chemotherapy may cause severe organ damage, worsening the infections and limiting access to transplant. Conventional gene addition or gene editing of autologous stem cells represent promising technologies that might overcome the limited number of available donors and offer a cure to all patients. Conditioning regimens based on monoclonal antibodies (mAb) specifically targeting hematopoietic stem and progenitor cells (HSPCs) are an attractive alternative to conventional myeloablation, in order to obtain depletion while preserving hematopoietic tissue homeostasis (32) (**Figure 1**). Overall, the development of these novel therapies will pave the way toward a new scenario of treatment to all RAG patients offering a cure regardless of the severity of the disease and age of treatment.

## Conventional Stem Cell Transplantation

Hematopoietic stem cell transplantation is the only curative treatment available for RAG deficiencies up to date (**Table 1**). Similarly to other SCIDs, highest survival is observed in patients transplanted before onset of infection and early in life (3.5 months of age or younger), regardless of donor or conditioning (43, 44). In this scenario, newborn screening is fundamental to expedite early treatment (45, 46).

**Table 1:** Main hematopoietic stem cell transplantation (HSCT) approaches and outcomes in recombination activating genes (RAG) patients.

N. Patients	Donor Source	Conditioning Regimen	Engraftment	Overall survival	IVIG	T cell reconstitution	Ref.
SCID including RAG defects	NA	Humanized anti-CD117 Monoclonal Antibody (AMG 191)	NA	2017-2027 ongoing	NA	NA	(33)
1 RAG1 OS	MMRD 4/6	anti-CD6 Pretreatment	No	Deceased at 5 months	NA	NA	(34)
3 RAG1 OS	MRD 6/6	No	Yes	Alive + 20yr	Yes in 40%	Yes	(34)
1 RAG1
1 RAG2	MUD	Pentostatin +	91%	Alive +1.5 yr	No	Yes	(35)
hypomorphic defect	T cell replete BMT	Low dose Cyclophosphamide + Busulfan Post-transplant Cyclophosphamide for GVHD prophylaxis	Myeloid chimerism				
1 RAG1 hypomorphic defect	HLA-haploidentical	Pentostatin + Low dose Cyclophosphamide + Busulfan Post-transplant Cyclophosphamide for GVHD prophylaxis	100% Myeloid chimerism	Alive +1.3 yr	No	Yes	(35)
1 RAG1 hypomorphic defect	MRD 6/10	G-CSF+Plerixafor	No	Alive +3.7 yr	Yes	Poor T cell counts	(36)
1 RAG1 hypomorphic defect	MRD 6/10	Alemtuzumab monotherapy	No	Alive +4.1 yr Second transplant MMUD (Cyclophosphamide-Melphalan)	Yes	Yes after 2 transplant	(37)
1 RAG1 OS	MUD 10/10	Alemtuzumab + anti-CD45 mAbs	94% myeloid chimerism	Alive +4 yr	No	Yes	(38)
48 RAG1 and 28 RAG2	MRD (25 pts)	No conditioning 3 received conditioning	Myeloid chimerism in 20% pts	85% + 2 yr	56% off IVIG	Yes in 50%	(39) Data refer to 145 Artemis and RAG pts
	MUD (7 pts)	Busulfan + cyclophosphamide	Yes	62%+2 yr	No	Yes	
	MMRD (5 pts)	No conditioning	No	46%+ 2 yr	Yes	Yes	
	HLA-haploidentical (39 pts)	Busulfan + cyclophosphamide/fludarabine	Myeloid chimerism in 77% pts	67%+ 2 yr	77% off IVIG	Yes in 93%	
		None or immunosuppression only	No myeloid chimerism	23% + 2 yr	Yes	Yes in 75%
8 RAG1 5 RAG2	HLA-haploidentical (4 pts)	11/13 conditioned with Campath ± Fludarabine and Melphalan or Busulfan + Cyclophosphamide	Variable myeloid chimerism	64.4%+ 10 yr	54% off IVIG	Yes in 50%	(40)
	MRD (7 pts)						
	mismatched						
17 RAG1/2	MRD	50% none 16% immunosuppression, 15% RIC, 46% myeloablative (different regimens including TBI, Bu, Cy, fluda, alemtuzumab, ATG),	Variable myeloid chimerism	97% + 5 yr	81% off IVIG	Yes in 76%	(41) Data refer to 240 SCID
	mismatched with/without conditioning			66% conditioning + 5 yr 79% no conditioning+ 5 yr	37% off IVIG	Yes in 66%	
	other			58%–74%+ 5 yr	70% off IVIG	Yes in 76%	
52 RAG1/2	MRD MMRD MUD	51% none 15% immunosuppression, 10% RIC, 22% myeloablative (different regimens including TBI, Busulfan, Cyclophosphamide, fludarabine, alemtuzumab, ATG, rituximab, anti-CD45 or other mAbs),	Variable myeloid chimerism	71%	NA	NA	(42) Data refer to 662 SCID

IVIG, intravenous immunoglobulin; SCID, severe combined immunodeficiency; NA, not applicable/not available; OS, Omenn Syndrome; MMRD, mismatched related donor; MRD, matched related donor; MUD, matched unrelated donor; MMUD, mismatched unrelated donor; BMT, bone marrow transplantation; yr, year; mAbs, monoclonal antibodies; pts, patients.

A study conducted in three main centers reported an 88% survival without recurrent infections in HLA-identical grafts without conditioning (39). Remarkably, half of the patients still required intravenous immunoglobulin (IVIG) and had incomplete T cell reconstitution, indicating that pre-transplant conditioning is necessary to eliminate immature progenitors. Conversely, patients receiving T-cell-depleted HLA-haploidentical family donors after myeloablative conditioning (busulfan combined with cyclophosphamide/fludarabine), had significant lower survival (63%) but showed higher T-cell reconstitution without the need for IVIG. Chronic GvHD and autoimmunity remained the most frequent late effects, observed in 24% of patients (39). The retrospective study from the Primary Immune Deficiency Treatment Consortium (PIDTC) confirmed poorer immune reconstitution in absence of conditioning, with ~80% overall survival (42).

Recently, a smaller cohort of 11 RAG patients receiving matched or haploidentical graft after pre-HSCT chemotherapy, showed similar overall survival (64.4%). Consistently, conditioning was associated with poorer overall survival and higher long-term side effects but better immune reconstitution (40). This observation has been reported in other SCIDs, as described in a large multicenter study on 240 SCID patients (including 17 RAG patients) (44).

Overall, myeloablative treatment can cause severe complications and mortality, while absence of pre-transplant conditioning may lead to poor immune reconstitution, due to competition with immature progenitors in bone marrow and thymic niches of RAG patients.

## Approaching Non-Genotoxic Conditioning for RAG Deficiencies

Although current conditioning regimens may cause acute and chronic toxicities, lymphoid progenitors limit proper engraftment of donor cells (11, 47–49), highlighting the urgent need for safe transplant protocols (**Table 1**). Biologic approaches based on mAbs specifically targeting HSPC while sparing non-hematopoietic cells are emerging as attractive conditionings for safely improving HSCT outcome. HSPC-depleting mAb as the anti-CD117 ACK2 (c-kit antagonist) allowed increased chimerism in *Rag2^-/-^γc^-/-^* mice, but not in immunocompetent mice (50) and X-linked chronic granulomatous disease (X-CGD) mice (51). Conversely, ACK2 synergistically acted with low-dose irradiation or CD47 blockade to allow higher engraftment in X-CGD (51) or immunocompetent mice (52), respectively. These data paved the way for the clinical trial using anti-CD117 antibody currently ongoing to treat SCID patients (NCT02963064) (33), which provides the proof of concept that a humanized mAb can safely clear human hematopoietic stem cells (HSC) niches facilitating donor cell engraftment in two T-B-NK+ SCID patients (with *Artemis* mutations) (53).

In the same direction, CD45 mAbs have been tested in a RAG1-deficient patient with immune dysregulation showing promising results (38). Alternative reduced intensity conditioning, including mAb combined with or without chemotherapy, has been tested in few RAG patients. The outcome of this treatment was variable, and some patients developed post-transplant severe complications (34–37, 54).

Antibody-Drug Conjugates (ADCs), extensively applied in cancer therapy, have recently proposed as non-myeloablative agents. CD117-ADC was exploited to deplete host HSPCs while preserving host immunity in immunocompetent mice (55), in MHC-mismatched allotransplantation (56) and in hemophilia A gene therapy (GT) mice (57). Encouraging results in non-human primates (58, 59) support ADC applicability in the clinical setting. In parallel, anti-CD45 mAb coupled with Saporin (CD45-SAP), a ribosome inactivating protein lacking the cell-entry domain and toxic only upon receptor-mediated internalization, has been exploited to efficiently deplete HSPCs enabling multilineage engraftment with minimal organ toxicity in immunocompetent mice (60). Due to CD45 expression pattern, CD45-SAP can be a good candidate in CID-G/AI patients to target autoreactive T cells, improving HSCT outcome and immune recovery. To this end, the efficacy of this compound was tested in null and hypomorhic *Rag1* models, achieving multilineage engraftment and robust immune reconstitution while preserving thymic epithelial cell homeostasis. A synergistic effect on myeloid chimerism and immune recovery was achieved when CD45-SAP was combined with low-dose of irradiation (61). However, mild transient hepatotoxicity secondary to CD45-SAP (60, 61) or upon single saporin injection (62) or radiolabeled anti-CD45 antibody (63–65) have been observed posing clinical concerns.

Overall, while these preclinical models indicate ADCs as safer conditional regimens than conventional chemotherapy, further investigation is needed before moving to the clinical setting. In particular, future studies assessing the dosage, safety, and efficacy of other HSC-depleting ADCs, alone or in combination with other mAb-based conditioning agents, are needed to bring ADC conditioning in the clinical arena.

## Gene Therapy of RAG Defects

The clinical spectrum of autoimmunity and hyper inflammation due to RAG mutations highlights the clinical need to offer a cure to patients without suitable donors or in critical clinical conditions.

Various groups have developed novel strategies, based on the hypothesis that gene corrected cells should acquire a selective advantage and overcome lymphocyte differentiation block. However, *RAG* tight regulation during cell cycle and expression level may constrain the clinical feasibility of GT. Ectopic or dysregulated *RAG* expression may lead to genotoxicity or immune dysregulation (66, 67). In the past, stable immune reconstitution in *Rag1^-/-^* mice was achieved using retroviral gene transfer of human RAG1 cDNA, but high transgene copy number was associated with a risk of lymphoproliferation (66). Based on this evidence, preclinical *Rag1^-/-^* models have been generated using self-inactivating (SIN) lentiviral vectors (LVs) carrying the human codon-optimized RAG1 cDNA (67–69) (**Table 2**). Different promoters have been tested to drive RAG expression: the human elongation factor 1α (EFS) promoter, the enhancer–promoter of the spleen-focus-forming virus (SFFV) and the ubiquitously acting chromatin opening element from the human HNRPA2B1-CBX3 locus (A2UCOE) (67, 73). Increased number and improved function of T cells were observed in the cohort of *Rag1^-/-^* mice transplanted with Lineage negative (Lin-) cells transduced with SFFV.coRAG1 and A2UCOE.coRAG1, with respect to EFS.coRAG1. However, poor B cell reconstitution was achieved with all promoters (68, 73). Despite reduced B cell number, GT mice showed increased production of IgM, IgG and IgA in the serum, antigen-specific antibody production upon challenges and polyclonal Vβ TCR repertoire. A parallel study using the same vectors reported contrasting results, showing inflammation, tissue cellular infiltrate and circulating anti-double strand DNA, resembling Omenn clinical features (67). Various factors may account for these discrepancies, including suboptimal transgene expression, partial immune reconstitution and immune dysregulation (73, 74). To improve LV titer and transduction efficiency, the Staal group switched to the CCL backbone, widely used in the clinics (69). They compared small and large scale production of the four SIN LVs carrying the following promoters: the phosphoglycerate kinase 1 promoter (PGK), the MND myeloproliferative sarcoma virus enhancer, the ubiquitous chromatin opening element (UCOE), Cbx3.MND (a tandem combination of UCOE and the MND promoter). The comparison identified the MND promoter as the optimal vector, able to reach a sufficient expression of the RAG1 transgene (with a vector copy number ~1) in order to obtain stable immune reconstitution of GT *Rag1* ^-/-^ treated mice. Despite low B cell number, gene therapy treated mice had normal Ig levels in the serum and showed a normalization of T cell specific antigen response. While polyclonal Vβ repertoire was restored, T cell counts in peripheral blood reached 30% of normal levels (69). Remarkably, in case of low RAG1 expression (mainly driven by PGK and UCOE promoters) 4 out of 9 GT mice developed skin erythroderma and wasting syndrome leading to death resembling observation previously reported in literature, further confirming the importance of achieving sufficient transgene expression. However, as previously discussed, irradiation and the expression of human RAG1 in mouse system may contribute to suboptimal immune reconstitution.

**Table 2:** Gene therapy preclinical studies in *Rag1* and *Rag2* mouse models.

Mouse Model	Vector(transgene)	T-cell Counts/Function	B-cell Counts/Function	Adverse events	Main conclusions	Ref.
*Rag1-/-*	MLV\-RV (RAG1)	Restored /Restored	Low /Restored	Undifferentiated acute leukemic proliferation (1/30 GT mice)	-Long-term correction -Immune reconstitution only with high VCN	(66)
*Rag1-/-*	EFS/SFFV /UCOE-SIN LV (RAG1 +/-co)	Improved /Restored	Low /Restored	Death due to BM failure in some GT mice	-Feasibility of SIN-LV-based correction -Critical importance of codon optimization	(68)
*Rag1-/-*	EF1a/SFFV/ PGK/UCOE/CP -SIN LV (coRAG1)	Very low /Reduced	Very low /Not fully restored	Autoimmunity (OS-like)	-Partial reconstitution and severe risk of adverse reactions with low VCN	(67)
*Rag1-/-*	Cbx3.MND/MND/PGK/ UCOE-SIN LV (coRAG1)	Improved in MND group /Restored in MND group	Low /Restored in MND group	Skin rashes and deaths in low co.RAG1 expressing mice (4/9)	-Crucial role of the promoter strength and co.RAG1 level for disease rescue -GMP-grade MND-LV for clinical testing	(69)
*Rag2-/-*	MLV-RV (RAG2)	Improved /Restored	Improved /Restored	Absent	-Long-term correction -Strong selective advantage	(70)
*Rag2-/-*	SF/UCOE /γcPr/RAG2p -SIN LV (coRAG2)	Improved in SF, UCOE groups /Restored in UCOE group	Improved in SF, UCOE groups	Undue death in 5/56 SF-treated mice (3/5 with leukemia)	-Immune function rescue with the UCOE.coRAG2 LV	(71)
*Rag2 R229Q*	UCOE (2.6/2.2Kb)-SIN LV (coRAG2)	Improved /Restored	Improved /Restored	Lymphoprolifera- tive thymic mass (1/35 GT mice)	-Improved immunodeficiency and autoimmunity in GT OS mice -*In vivo* variability correlated to transduction levels	(72)

BM, bone marrow; CP, cell type-restricted promoter; co, codon optimized; EFS or EF1α, elongation factor 1 α; γcPr, γ chain promoter; GT, gene therapy; LV, lentiviral vector; MLV, Moloney leukemia virus; MND, myeloproliferative sarcoma virus enhancer, negative control region deleted, dl587rev primer-binding site substituted; OS, Omenn Syndrome; PGK, phosphoglycerate kinase 1 promoter; RAG2p, RAG2 promoter; RV, retroviral vector; SIN, self-inactivating; SFFV or SF, spleen-focus-forming-virus; UCOE, ubiquitously acting chromatin opening element; VCN, vector copy number.

Different LVs were also tested in CD34+ cells from RAG1 patients to test their ability to induce the differentiation of functional B and T cells *in vivo* in NSG mice. In a first report, *Rag1* expression driven by EF1α allowed the differentiation of functional B cells in a minority of transplanted mice, while no information on T cells are reported (75). Recently, LV carrying MND promoter was used to transduce CD34+ cells from a hypomorphic RAG1 patient with residual B cells and no T cells, transplanted in a single NSG mouse. CD34+ GT cells could differentiate in polyclonal B cells and T cells, despite the low vector copy number, in line with the stronger MND promoter activity (69).

GT studies in hypomorphic *Rag1* models will be instrumental to understand the efficacy of GT in atypical SCID or OS, which represent the majority of RAG patients. Finally, safety tests and long-term follow up of GT treated animals are required to further validate the use of RAG1 GT in the clinical setting.

With regard to RAG2, preliminary studies using a retroviral vector carrying human RAG2 cDNA showed stable immune reconstitution in the absence of detectable toxicity (70). Because of the genotoxicity of retroviral vectors (76, 77), a SIN LV carrying human codon optimized RAG2 cDNA driven by UCOE promoter has been developed showing promising data in the preclinical model of *Rag2* ^-/-^ mice in terms of immune reconstitution. GT *Rag2* ^-/-^ mice presented polyclonal Vβ TCR repertoire, increase of naïve T cells and redistribution of T cell subpopulation. Reduced B cell counts were accompanied by normal levels of IgM, IgG subclasses and antigen-specific antibodies production upon T-dependent and independent antigens (71).

Based on these promising results, the UCOE-RAG2co LV has been tested in the mouse model of OS, the *Rag2^R229Q/R229Q^* mutant (26). While GT OS mice showed decreased absolute T and B absolute counts as compared to wild-type mice, significant change in T cell distribution with a dramatic increase in naïve T cells and reduction in effector/memory T cells was obtained (72). Treated thymic displayed improvement in the structure with appearance of medullary compartment containing mature TEC expressing AIRE. Consistently, spectratyping indicated a polyclonal T cell repertoire. Finally, gene corrected mice responded properly to *in vivo* challenges. Remarkably, treated OS animals did not show cellular infiltration in the skin and gut indicating a resolution of Omenn clinical signs. Taken together, these data indicate the feasibility of lentiviral GT for RAG2 deficiencies, even in the context of residual recombination activity and in inflammatory conditions.

## Conclusions and Future Directions

Autologous gene corrected stem cell transplant represents the next therapeutic step to treat RAG patients without suitable donors. Preclinical RAG models have provided instrumental data unrevealing advantages and drawbacks of novel cell-based therapies and non-genotoxic conditioning posing the basis for the future development of clinical GT trials.

HSPC expansion protocols assuring maintenance of stemness potential are currently being tested when limited donor cells are available, especially in case of cord blood donors (78, 79). Notably, these protocols can be exploited to decrease the burden of CD34+ cell harvest in very young patients in gene therapy and editing settings (80). Conventional GT approach has shown promising results in murine models, highlighting at the same time the need for physiological level of RAG expression while maintaining low vector copy number. Preclinical GT studies are now in progress to implement transgene expression. To this end, the recent description of the effect of immunomodulatory compounds is particularly relevant, leading to increased transduction levels in long-term HSC while preserving engraftment potential (81–83). Cyclosporines A and H have been recently demonstrated improve transduction without altering HSCP subpopulation composition nor the cell-cycle status (81, 82). Prostaglandin E2 (PGE_2_) allows enhanced transduction efficiency in one hit protocol thus limiting HSPC manipulation (83, 84). Consistently, PGE_2_ has been recently applied in a GT phase I/II clinical trial to treat mucopolysaccharidosis type I, Hurler Syndrome (85).

Although currently still challenging, genome editing at a specific locus is now emerging as a new potential technique that allows to insert the corrective sequence downstream its endogenous promoter thus maintaining the physiological expression of the gene of interest (86–89). The development of engineered nucleases, including Zinc Finger Nucleases (ZFNs), transcription activator like effector nucleases (TALEN) and clustered regularly interspaced short palindromic repeats (CRISPR-Cas9), associated with improvement in the efficiency of homology directed repair in HSCs (90) will allow to obtain rapid advances for the future transfer of this technique to the clinical setting.

## Author Contribution

AV, VC, and MC designed and wrote the manuscript. All authors contributed to the article and approved the submitted version.

## Funding

This work was sponsored in part by EU H2020 grant RECOMB (755170-2), Telethon Core grant E2 project, RF-PE- 2016-02363691 and PRIN 2017 5XHBPN.

## Conflict of Interest

The authors declare that the research was conducted in the absence of any commercial or financial relationships that could be construed as a potential conflict of interest.
